# 

**Published:** 1898-06

**Authors:** 


### CONTENTS:

### SELECTIONS.

Article I.—Dr. A. J. Volck's Seventieth Birthday. ............... 49

" II.—A Discussion Following the Reading of a Paper upon "Syphlitic Affections of the Mouth. Dr. H. H. Burchard. ............... 63

" III.—Prosthesis. Wm. Ernest Walker, D. D. S............ 72

" IV.—The Relative Value of Fillings. Dr. C. P. Pruyn.... 75

" V.—Physiological Testing of Drugs. ............... 77

" VI.—Hydronaphthol—With Suggestions for Its Use in the Treatment of the Dental Pulp, Alive or Dead. Sidney S. Stowell, D. D. S............... 79

" VII.—Dentists' Convention ............... 85

" VIII.—Caoutchouc and Gutta-Percha Cements.......... 88

### EDITORIAL, ETC.

Dr. A. J. Volck's Seventieth Birthday. A Characteristic Event... 90

Remarks of Dr. E. E. Cruzen............... 92

State Dental Association............... 92

### MONTHLY SUMMARY.

A New Function of the Antrum ............... 93

Floss Silk............... 94

Filling Materials............... 95

Danger from Air Injection............... 96

